# Immune Escape Strategies of Malaria Parasites

**DOI:** 10.3389/fmicb.2016.01617

**Published:** 2016-10-17

**Authors:** Pollyanna S. Gomes, Jyoti Bhardwaj, Juan Rivera-Correa, Celio G. Freire-De-Lima, Alexandre Morrot

**Affiliations:** ^1^Departamento de Microbiologia Geral, Universidade Federal do Rio de JaneiroRio de Janeiro, Brazil; ^2^Division of Parasitology, Council of Scientific and Industrial Research-Central Drug Research InstituteLucknow, Uttar Pradesh, India; ^3^Academy of Scientific and Innovative ResearchAnusandhan Bhawan, New Delhi, India; ^4^Division of Parasitology, Department of Microbiology, New York University School of MedicineNew York, NY, USA; ^5^Instituto de Biofísica Carlos Chagas Filho, Universidade Federal do Rio de JaneiroRio de Janeiro, Brazil

**Keywords:** plamosdium, evasion strategies, liver stage, blood stage, malaria, microbiology, immulogy

## Abstract

Malaria is one of the most life-threatening infectious diseases worldwide. Immunity to malaria is slow and short-lived despite the repeated parasite exposure in endemic areas. Malaria parasites have evolved refined machinery to evade the immune system based on a range of genetic changes that include allelic variation, biomolecular exposure of proteins, and intracellular replication. All of these features increase the probability of survival in both mosquitoes and the vertebrate host. *Plasmodium* species escape from the first immunological trap in its invertebrate vector host, the *Anopheles* mosquitoes. The parasites have to pass through various immunological barriers within the mosquito such as anti-microbial molecules and the mosquito microbiota in order to achieve successful transmission to the vertebrate host. Within these hosts, *Plasmodium* species employ various immune evasion strategies during different life cycle stages. Parasite persistence against the vertebrate immune response depends on the balance among virulence factors, pathology, metabolic cost of the host immune response, and the parasites ability to evade the immune response. In this review we discuss the strategies that *Plasmodium* parasites use to avoid the vertebrate host immune system and how they promote successful infection and transmission.

## Introduction

Malaria still remains one of the biggest global health burdens and causes of mortality in the world. Malaria mainly occurs in tropical and subtropical parts of the world, being a serious cause of mortality and morbidity in Sub-Saharan Africa. There are five species of *Plasmodium* that cause disease in humans: *P. ovale, P. malariae, P. vivax, P. falciparum*, and the primate species *P. knowlesi*. In a previous report by the WHO, there were 215 million cases and 438 thousand deaths due to malaria in 2015 (WHO, 2015). Malaria is transmitted by the female *Anopheles* mosquito during a feeding. As the mosquito feeds on its host, sporozoites are released into the blood stream, and they migrate to the liver (Cirimotich et al., 2010). The complete life cycle of *Plasmodium* requires two hosts: the mosquito vector and the vertebrate host (Crompton et al., 2014). In the vertebrate host it undergoes two stages: an asymptomatic pre-erythrocytic stage (liver stage), and a symptomatic erythrocytic stage (blood stage) (Haque and Engwerda, 2014). The journey of sporozoites to the liver is asymptomatic (Zheng et al., 2014), but various reports have described that an initial immune response occurs during this stage. Some of the immunological responses described to occur during the liver stage are: apoptosis of infected cells (Meslin et al., 2007), isolation and targeting of parasites in specific compartments for elimination (Yano and Kurata, 2011), type I IFN production induced by parasite RNA (Liehl et al., 2015) and LC3-mediated autophagy targeting of sporozoites (Risco-Castillo et al., 2015). Nevertheless, several mechanisms such as immune evasion, immune exploitation, and molecular piracy are employed by the parasites to promote their survival in the host (Hisaeda et al., 2005). After the first immunological attack during liver stage, the majority of parasites that survived will replicate within the hepatocytes and amplify their number exponentially leading to the release of hundreds of thousands of merozoites into circulation (Prado et al., 2015). The released merozoites will subsequently initiate the symptomatic blood stage cycle, which is responsible for the clinical features and pathologies associated with malaria (Ocaña-Morgner et al., 2003).

During the blood stage some parasites will differentiate into male and female gametocytes. The gametocytes will be ingested by other feeding mosquitoes where they will further develop inside the mosquito gut (Cirimotich et al., 2010). Within the mosquito, ineffective sporozoites will be kept until the next blood meal, where they will be transmitted to a mammalian host continuing the parasite life cycle (Molina-Cruz et al., 2012). The complexity of the parasite life cycle allows for the use of various immune evasion strategies by *Plasmodium*, which pose a challenge to the development of a malaria vaccine (Hisaeda et al., 2005). In this review, we discuss some of the immune evasion strategies that have been described for *Plasmodium* at both the liver and the blood stages. We also highlight how these immune evasion strategies can promote successful infection, overcoming many of the challenges that human pathogens face with the host immune response.

## Immune evasion during the pre-erytrocytic stages

### Mechanical strategies to overcome the first barrier: the skin

An infected mosquito bite transmits around 100–200 sporozoites in the human skin and despite innate immune destruction of most of them; some parasites are still able to establish successful infection at very low numbers (Risco-Castillo et al., 2015). The skin is the first barrier that parasites encounter after transmission into the vertebrate host (Cirimotich et al., 2010). Thus, sporozoites have evolved to overcome this barrier by various mechanical strategies such as motility and cell transversal (Tavares et al., 2013). To achieve this, sporozoites are equipped with specialized mechanical proteins that help them achieve successful passage. This has been evidenced by studies that show how the transmission of sporozoites deficient in SPECT-1 (spororozoite microneme protein essential for cell traversal) and SPECT-2 (also called perforin-like protein 1 or PLP1) are blocked in the dermis and are ingested by phagocytes preventing their progression. These proteins were reported to be necessary for cell traversal and for migration to the liver (Patarroyo et al., 2011). The sporozoites can tranverse various kind of cells, wich includes immune cells. The transversal of immune cells can lead to inactivation of immune cell defenses and prevent the clearance of sporozoites (exocytosis) before crossing the barrier (Sinnis and Zavala, 2012). Another protein responsible for motility of sporozoites is TRAP (Thrombospondin-Related Anonymous Protein). This protein is found on the micronemes and the surface of sporozoites. TRAP allows the parasite to interact with surface host molecules that provide gliding motility to exit the dermis. Additionally, TRAP can bind to sulfated glycoconjugate motifs that can aid in recognition and entry to hepatocytes (Müller et al., 1993). Regardless, some of the sporozoites will enter the lymphatic system where they can be recognized and destroyed by immune cells such as dendritic cells (DCs) (Wilson et al., 2016).

### Overcoming the hepatic immune defenses to establish liver stage infection

A successful infection in great part is due to the parasite evading the first attacks of the host immune response, that despite its strength, it is not enough to block the development of the blood stage (Singh et al., 2010). After the sporozoites migrate from the skin, crossing all cellular barriers, they will reach the blood, and consequently the lymphatic system (Crompton et al., 2014). Once inside the circulatory system, sporozoites rapidly reach the sinusoid cavity of the liver. The exoerythrocytic (liver) stage has been described as asymptomatic because the liver is an immunoprivileged organ that is protected against a strong immune response (Liehl et al., 2015). Even though initially the liver stage was considered an unresponsive phase, various reports have showed that the liver immune cells are present and active in different rodent malaria models, suggesting an initial immune response during the liver stage (Liehl et al., 2014). Some reports have described how *Plasmodium* RNA could activate the type I interferon (IFN) pathway via the cytosolic Pattern Recognition Receptor (PRR), MDA5 (Liehl et al., 2014). Type I interferons are potent inflammatory cytokines that are known to inhibit growth of exoerythrocytic forms (Hisaeda et al., 2005). Some of the cells that mediate these anti-parasitic effects include Natural Killer and Natural Killer T cells (NK, NKT), and γδT cells. These cells mainly inhibit parasite growth through secretion of interferon, including both type I interferons and IFN-γ (Ocaña-Morgner et al., 2003; Risco-Castillo et al., 2015). Other molecules such as hepcidin have been implicated in growth inhibition of exoerytrhrocytic phases (Spottiswoode et al., 2014).

### Modulation on of Kupffer cells

To invade hepatocytes, sporozoites must cross the barrier lined by endothelial cells (ECs) and immune phagocytic cells called Kupffer Cells (KCs) (Tavares et al., 2013). Sporozoites will have to interact with these resident cells in order to establish a successful infection (Meslin et al., 2007).

When the sporozoites reach the liver they are attracted to sulfated molecules that are present in ECs and KCs. This interaction is mainly mediated between the circumsporozoite protein (CSP) and sulfated heparin sulfate proteogycans (HSPGs) in the surface of the host cells. Other molecules that are involved in this process include P39 and CD38 (Cha et al., 2015). Previous studies employing intravital and electron microscopy suggested KCs, but not the ECs are the preferred passage used by sporozoites (Cha et al., 2015). Recent studies proposed that sporozoites exploit multiple paths to cross the sinusoidal barrier. Also, there have been reports that sporozoites pass through gaps between ECs and KCs, not engaging in traversal capacity (Meis et al., 1983). For the sporozoites passing through KCs, it would be interesting to know why the KCs do not degrade parasites even though they efficiently kill other microorganisms that invade hepatocytes (Sinnis and Zavala, 2012). Additionally, it has been described in a mice model that sporozoites can go through KCs and modulate their cytokine profile, leading to the down-regulation of Th1 cytokines (TNF-α, IL-6, and MCP-1) and the upregulation of Th2 cytokines (IL-10) for ensuring safe passage (Klotz and Frevert, 2008). Additionally, CSP can interact with LRP-1 (low-density lipoprotein receptor-related protein) and proteoglycans on the KC surface, which increases the levels of intracellular cAMP/EPAC and prevents the formation of reactive oxygen species (ROS). ROS is a natural byproduct that is produced during environmental stress and can cause cellular damage and it can kill the parasite (Ikarashi et al., 2013). In some cases, the parasite forces the KCs into apoptosis. Lastly, sporozoites can also negatively affect the antigen-presentation capacity of KCs, which display reduced expression of MHC- class I and IL-12 (Steers et al., 2005). Some evidence suggests that sporozoites are able to manipulate the KCs functions and immunosupress the microenvironment for its own advantage.

### Modulation of hepatocytes

After successful penetration of the sinusoidal cell layer, sporozoites enter the hepatocytes and start intra-hepatic development. Sporozoites actively invade the host cells (hepatocytes) employing the cholesterol uptake pathway, which is different from other microorganisms that exploit phagocytic activity of host cells for invasion (Itoe et al., 2014). Additionally, released CSP supports parasite development through the suppression of the NF-κB signaling pathway (Ding et al., 2012) and the up regulation of host heme oxygenase-1 (HO-1), which further promotes the parasite development in the liver by modulating the host inflammatory response (Pamplona et al., 2007). Sporozoite infection of hepatocytes also interferes with the mTOR pathway, which alters the levels of proteins involved in cell survival, proliferation, autophagy, anabolism, and cell growth (Hanson et al., 2013). After invading the final hepatocyte, sporozoites are enclosed in a parasitophorous vacuole (PVM), which physically separates it from the host cytoplasm thereby avoiding degradation by the endocytic/lysosome system. This isolation keeps the parasitophorous vacuole isolated from cell intrinsic defenses such as apoptosis and selective autophagy (Thieleke-Matos et al., 2016). The transition of parasites from liver stage merozoites to initiate blood stage represents another key point of the cycle for immune evasion. In order to start the blood stage, liver stage parasites must exit from the hepatocytes through hepatic spaces where they are exposed to resident phagocytic cells such as KCs and DCs. Merozoites avoid being killed by liver phagocytes by covering themselves in membranes derived from the host known as merosomes (Sturm et al., 2006). The merosomes are derived from infected hepatocytes when they bud off from the cells and manage to avoid recognition by phagocytes to initiate blood stage (Garg et al., 2013). The initiation of blood stage could take days after exiting the liver. Collectively, all of this data demonstrates the long journey that parasites travel to overcome the immune system.

## Immune evasion by erythrocytic parasite stages

The blood stage is where the clinical symptoms that are characteristic of malaria appear. Blood stage is initiated when merozoites released from hepatocytes invade RBCs to develop into ring shaped, and young and mature trophozoites, undergoing schizogony. In schizogony each one leads to six to 32 daughter clones that will burst out into the bloodstream and reinvade new RBCs (Garg et al., 2013). An effective anti-parasitic immunity has been proven to be complex and involving various elements of the immune system. Antibodies and T-cells have proven to be important components in achieving parasite clearance (Chotivanich et al., 2002). Antibodies can have anti-parasitic activity by binding to infected erythrocytes and mediating their phagocytosis (opsonization) by circulating macrophages (Dups et al., 2014). They can also prevent invasion of red blood cells by binding to extracellular merozoites and marking them for clearance or lysis by complement. T-cells play an essential role by both direct and indirect mechanisms. Direct mechanisms include production of pro-inflammatory cytokines such as IFN-γ and TNF-α that prime macrophages and other components of the anti-parasite immune response (Nasr et al., 2014). They also mediate an indirect mechanism by activating specific B-cell clones that will produce anti-parasite antibodies (Kafuye-Mlwilo et al., 2012). Other innate immune components that have been attributed to play a role include: NK cells, γδT cells (Inoue et al., 2013), host microbiota and natural antibodies (Crompton et al., 2014). Circulating infected blood cells can be targeted for destruction in the spleen but the parasite avoids this by developing several mechanisms to evade the host immune response (Chotivanich et al., 2002). As an example for complement evasion, inhibition of the membrane attack complex (MAC) formation has been described in *P. falciparum*-infected patients and it even correlated with severe malaria cases (Schmidt et al., 2015).

The intracellular survival is the most primitive immune escape mechanism of parasites, which avoid the direct interaction of parasites with the immune cells. Furthermore, RBCs do not express MHC class I molecules on their surface thereby escaping from recognition by CD8^+^ T cells (Bowen and Walker, 2005). Another feature that parasites use to avoid clearance is the formation of rosettes. Rossettes is a phenomenon where infected erythrocytes cluster with uninfected erythrocytes helping the parasites bind to RBC epitopes and avoid immune recognition. Blood type is one factor correlated with the rosette formation. For example parasites that bind blood type A are more virulent than the ones that bind blood type O because their capacity of forming rosettes is stronger (Moll et al., 2015).

Overall the evasion mechanisms are divided in two fields of strategies. One of them is the expression of variable antigenic proteins at the surface in different life cycle stages of parasites that help camouflage them from the host immune system (Escalante et al., 1998). The evasion of immune clearance is due the highly polymorphic proteins that mediate antigenic variation by changing and adapting to host immune response, promoting long-lasting infections (Wilson et al., 2016). The second is sequestration, which is mediated by genes products of the *PfEMP-1, Var, Rifin* (Mwakalinga et al., 2012), and *Stevor* multigene families (Kraemer and Smith, 2006). These proteins allow iRBC adherence in vascular endothelium hence avoiding clearance, and sequestering them in the microvasculature of various organs. They also exploit host components such as platelets and inflammation that can lead agglutination of uninfected RBCs with iRBCs and promote the appropriate sequestration microenvironment (Helmby et al., 1993).

In the blood stream, merozoites utilize complex set of proteins in order to invade uninfected red blood cells (Helmby et al., 1993). The parasite locates closely its proteins to proteins of the host erythrocytes, mediating reorientation and tight binding allowing them to invade the host RBCs. MSP-1 (merozoite surface proteins) anchors in the host erythrocyte membrane through GPI (glicosilfosfatilinositol) anchors. (Nosjean et al., 1997). MSPs along with other merozoite proteins called erythrocyte binding-like (EBL) proteins exist in several alleles or copies in the genome, showing a high degree of polymorphism (Holder et al., 1999). Several studies about new vaccine candidates against malaria are based in interactions between surface proteins, such as MSPs, GPI and PfEMP-1 (Boyle et al., 2014). The EBL protein family has a sophisticated profile with redundant functions that evolved to promote antigenic variation and immune evasion (Souza-Silva et al., 2014). EBL proteins are proteins that contain a Duffy-binding-like (DBL) which have been shown to mediate host receptor binding, in the case of *P.vivax* infection (VanBuskirk et al., 2004).

All *Plasmodium* species use to the same principle for invasion of the cell through interaction of parasite proteins to host erythrocyte receptor. The most studied invasion mechanisms are the ones used by *P.vivax* and *P.falciparum*. The merozoites of *P.falciparum* have at least five mechanisms of invasion, characterized by its respective receptor in erythrocytes (Baum et al., 2003). *P.vivax* shows great preference to invade immature red blood cell forms, such as reticulocytes. Some studies have reported that people who have the DARC (Duffy antigen receptor chemokines) in erythrocytes are more susceptible to infection by *P. vivax*. Conversely, the lack of this receptor (Souza-Silva et al., 2014) confers resistance to infection by *P. vivax*. However, other studies from west Kenya and Brazil have reported *P.vivax* cases, despite the lack of DARC expression in RBCs, suggesting alternative pathways for *P.vivax* infection (Ryan et al., 2006).

*P. falciparum* has evolved more adapt ways of immune evasion. One of the most studied strategies is the exposure PfEMP1 on infected red blood cells. The PfEMP-1 expression is a sophisticated apparatus that promotes the ability to bind different host endothelial cells, such as human brain microvascular endothelial cells (HBMEC). This binding helps the parasites avoid clearance by the spleen and it‘s directly related to complicated falciparum malaria such as cerebral malaria (Abdi et al., 2016).

The alterations in the iRBC membrane created by exposure of these proteins are denominated Knobs. These knobs will mediate cytoadhesion to the endothelium. Some of the targeted endothelium receptors include EPCR, CSA, CD36, and ICAMs (Yipp et al., 2000). Condroitin sulfate A (CSA) is the receptor targeted by the specific variant that leads to placental malaria in pregnant women (Kraemer and Smith, 2006). Each PfEMP variant can promote binding to different receptors in several organs (Hviid and Jensen, 2015). In both iRBC and uninfected RBCs in severe malaria, the erythrocytes become rigid following impediment to their flow through capillaries that have midpoint diameters that are all smaller than the erythrocyte itself (Dondorp et al., 2000). This can block parasite recirculation or recruitment of non-infected RBCs leading to rosettes (Uyoga et al., 2012) and has been associated with severe malaria anemia (Uyoga et al., 2012). Knobs sequestration in post-capillary venules occurs in mature blood stages such trophozoite and schizonts (Sharma, 1991). The presence of the parasite leads to activation of the immune response and production of cytokines, enhancing expression of receptors in endothelial cells targeted by the parasite adhesins (Rowe et al., 2009). This promotes the cyclical fevers associated with malaria while the constant presence of parasite exacerbates the inflammatory immune response (Gazzinelli et al., 2014).

### Inadequate innate and adaptive responses against *Plasmodium* blood stage parasites

Even though the parasite has evolved various immune evasion strategies, the host immune response along with its genetic background, are essential for parasite control and prevention of clinical malaria. The human immune system is equipped with both innate and adaptive responses with great anti-parasitic activity. One major component of the innate immune system are Patter Recognition Receptors (PRRs) that recognize Pathogen-associated molecular patterns (PAMPs). In the case of malaria, three PAMPs that have been proposed for Plasmodium parasites are: GPI anchors, Hemozoin and immunostimulatory nucleic acid motifs (Gazzinelli et al., 2014).

Release array of pro and anti-inflammatory can result in either successful parasite control or alternatively an exacerbated immune response leading to pathology (Perkins et al., 2011). Even though mounting a potent immune response is not always completely efficient, it is necessary for controlling parasitemia. An important “bridge” between the innate and adaptive immune response are the dendritic cells (DCs). The DCs have a function of antigen presentation, stimulation of T-cells, and are major mediators of the adaptive immune response (Gowda et al., 2012). During malaria, CD4+ T− helper cells have been implicated for pathogenesis, protection and immune evasion of parasites (Wykes et al., 2014). The role of regulatory T cells (expressing FOXP3 CD4+ CD25+) in immunity has been controversial. For example, lack of T-regs during experimental cerebral malaria mice models renders them more susceptible to disease; while in human cerebral malaria correlates with higher parasitemia (Walther et al., 2005).

The role of CD8+ T cells-mediated immunity in blood stage has been less studied. This is mainly due to the fact that iRBCs don't express MHC class I rendering them resistant to the cytotoxic effect of CD8+ T-cells. Additionally, the high-level of mutations of epitopes in blood stage lead to immune evasion of the cytotoxic T lymphocyte (CTL) response, and hence fail to generate highly effective malaria vaccines (Templeton, 2009). Regardless, experiments using depletion both CD8+ and CD4+ T-cells during blood stage of *P.chabaudi* infections in mice resulted in a delayed clearance of the infection (Podoba and Stevenson, 1991), suggesting a possible role for CD8+ T-cells during blood stage malaria.

B cells have been attributed to an essential protective role during malaria. Antibody responses as immune agents alone can provide sufficient protection to control clinical disease (Wykes et al., 2014). The functions of antibodies can act on limiting the growth of blood stage parasites and the development of clinical symptoms for several mechanisms, like blocking erythrocyte invasion (Blackman et al., 1990), act as opsonins on parasitized erythrocytes (Osier et al., 2014), monocyte-mediated antibody-dependent cellular killing (Bouharoun-Tayoun et al., 1995), and complement-mediated lysis (Boyle et al., 2015), as well as interfering with the adherence of infected erythrocytes to vascular endothelium (Beeson et al., 2004). Unfortunately, the antibody responses to malaria infection from children and adults have been reported to be short-lived and rapidly lost in the absence of continued parasite exposure (Ryg-Cornejo et al., 2016). Collectively, these findings demonstrate that the immune system is efficient at reducing parasite burden but in most cases is not enough to prevent the progress of disease showing the high capacity of immune evasion by the parasite.

## Concluding remarks

*Plasmodium* has evolved a range of biomolecular strategies in order to escape the immune response and to guarantee the survival within the host. These parasites have great immune evasion ability across their whole life cycle. From an asymptomatic liver stage to a sophisticated system of proteins (such as PfEMP proteins) that is utilized by the parasite to avoid immune recognition and establish a successful infection. Hence further studies requiring close interactions of biomolecular, genetic, and immune strategies in both host and parasites are needed in order to understand better a protective anti-Plasmodium immunity. Thereby, in this review we summarize some of the important mechanisms that Plasmodium parasites utilize to facilitate evasion of the vertebrate host immune system across its life cycle. The challenges for the development of more effective therapeutics will have to overcome the various parasite immune evasion strategies and as several species cause malaria, several antigenic variations have to be considered. Recently we have a good candidate malaria vaccine in phase III (RST,S - GSK) of implanting, but even with a good initial response, it was also proved to be incomplete in points of recurrence of the disease (Morrison, 2015). Thus, the well-studied avoidance schemes propose an opening of new possibilities for further studies to consolidate the burden that malaria causes to hundreds of thousands of people annually.

## Author contributions

PG, JB, JR, CF, and AM wrote the paper. All authors read and approved the final version of the manuscript.

### Conflict of interest statement

The authors declare that the research was conducted in the absence of any commercial or financial relationships that could be construed as a potential conflict of interest.
